# Supplementary material for: N6-Methyladenosine Directly Regulates CD40L Expression in CD4+ T Lymphocytes
Source: Biology (Basel). 2023 Jul 14;12(7):1004. doi: 10.3390/biology12071004 (PMC10376055; doi:10.3390/biology12071004)
Supplement: Supplementary file 1 [file biology-12-01004-s001.zip › biology-2419798-supplementary.pdf]

Supplemental information

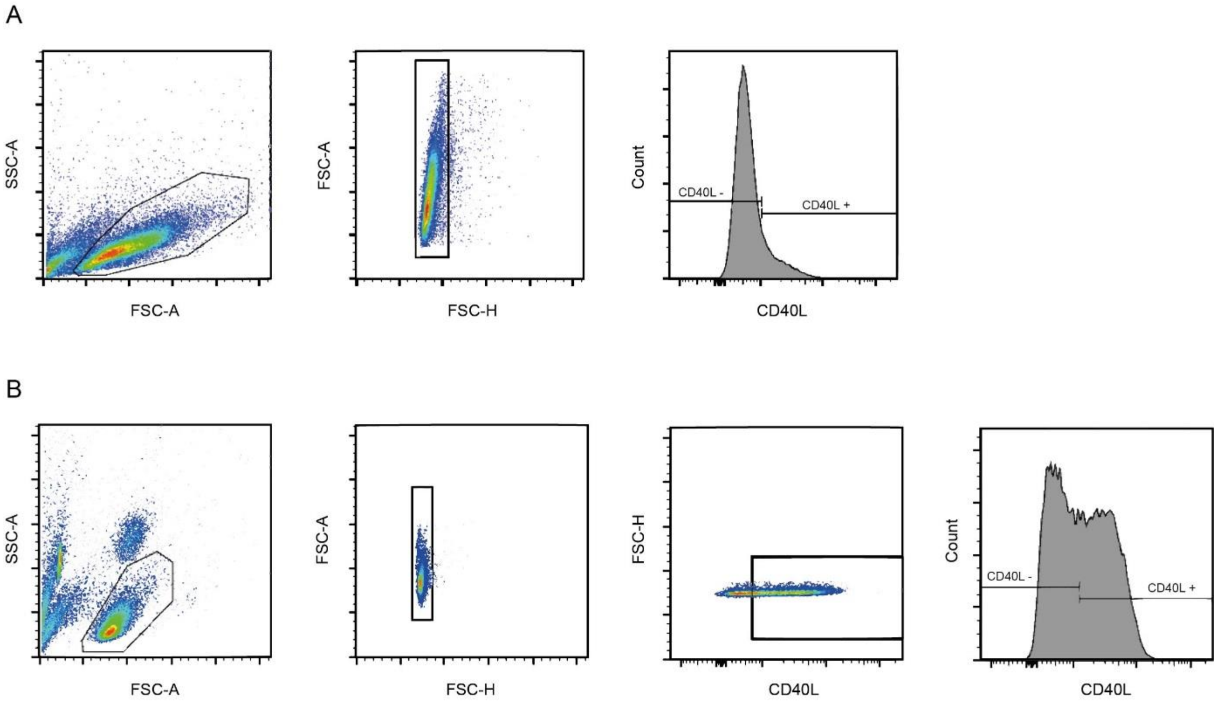

Figure S1. CD40L expression is regulated via m^6A 'eraser' FTO in activated CD4$^+$ T lymphocytes. (a) Jurkat cells with CRISPR-Cas9 knockout of FTO were activated with 4 hours PMA and ionomycin. Flow cytometry gating strategy. **(b)** Human CD4$^+$ T lymphocytes were pre-incubated for 48 hours with 50µM entacapone. The cells were subsequently activated with CD3/CD28 beads overnight. Flow cytometry gating strategy.

Table S1 – qPCR primers

Target gene	Forward (5′ to 3′)	Reverse (5′ to 3′)
CD40L	ATGGGAAACAGCTGACCGTT	GATTGTTGCCCGCAAGGTTT
Pre-CD40L	CCTCCTCTTGTTGATGCCGT	AGGATCTTTCTCCTGTGTTGCAT
β2M	GGCATCTTCAAACCTCCATG	ATGAGTATGCCTGGCCGTGTGA
GUSB	AGACAAGGGGGCTCCGTA	CGTTTCTGCTCCATACTCGC
SETD7	GGGGTTCAGAGACCTGGAAT	GCATGGTGAGAGGATGTGAC

Table S2 – Flow cytometry antibodies

Marker	Fluorochrome	Catalog number	Company
Fixable viability dye	eF506	15560607	Invitrogen eBioscience
Mouse anti-CD3	APC/Cy7	2102130	Sony Biotechnology
Mouse anti-CD4	PerCP/Cy5.5	300530	Biolegend
Mouse anti-CD40L	PacB	310820	Biolegend

Table S3 – Western blot antibodies

Target gene	Source	Catalog number	Company
FTO	Rabbit	ab124892	Abcam
H3	Rabbit	9715S	Cell Signaling Technology
Anti- rabbit HRP	Swine	P021702-2	Agilent
Anti- mouse HRP	Rabbit	P026002-2	Agilent
